# Hemodynamic Assessment via Echocardiography During Propofol Anesthetic Induction in Healthy Dogs

**DOI:** 10.1155/2024/5541917

**Published:** 2024-10-12

**Authors:** Thais Cabral de Oliveira, Guilherme Andraus Bispo, Laura Beatriz de Socorro Poleto, Francisco Dantas de Maio Martinez, Max Túlio Rocha de Souza, Lais Calazans Menescal Linhares, Marilda Onghero Taffarel, Wagner Luís Ferreira, e Paulo Sérgio Patto dos Santos

**Affiliations:** ^1^São Paulo State University (UNESP), School of Veterinary Medicine of Araçatuba (FMVA), Araçatuba, São Paulo, Brazil; ^2^São Paulo State University (UNESP), School of Agricultural and Veterinary Sciences (FCAV), Jaboticabal, Brazil; ^3^São Paulo State University (UNESP), School of Veterinary Medicine and Zootechnism (FMVZ), Botucatu, Brazil; ^4^State University of Maringá (UEM), Department of Veterinary Medicine, Umuarama, Paraná, Brazil

**Keywords:** heart rate, hemodynamics, myocardial function, stroke volume, TIVA

## Abstract

**Introduction:** Propofol is an intravenous anesthetic administered as a bolus or continuous infusion during anesthetic induction and maintenance. Its pharmacokinetic characteristics include hepatic and extrahepatic metabolism with a rapid onset of action and short duration, which provides a smooth anesthetic induction without excitatory effects.

**Objective:** To evaluate whether the isolated use of propofol in anesthetic induction in dogs changes the hemodynamic variables assessed via echocardiography.

**Study Design:** Prospective clinical study.

**Animals:** Twelve healthy dogs.

**Methods:** The dogs were induced with propofol (dose/effect) at 3 mg/kg/minute, and echocardiographic evaluations were performed immediately before anesthetic induction (MB) and immediately after its interruption (MI), at the end of the supply of the anesthetic agent.

**Results:** A significant reduction was observed between the values of the following hemodynamic variables: Ejection Fraction (EF%), which varied from 70% to 65% (*p*=0.011) between moments, and the Doppler Ejection Index (DEI), which ranged from 27.1 mL/beat/m^2^ to 22.4 mL/beat/m^2^ (*p*=0.044). The heart rate (HR) and the other studied hemodynamic variables showed no significant differences between the evaluated moments.

**Conclusion and Clinical Relevance:** Propofol was a safe anesthetic-inducing agent, maintaining stable hemodynamic indices during anesthetic induction at the used rate.

## 1. Introduction

Propofol, an intravenous anesthetic from the alkylphenol family, is widely used as a sedative-hypnotic agent in veterinary medicine and is often combined with ultra-short-acting opioid analgesics, lidocaine, and/or ketamine [1, 2]. It has a hypnotic potency approximately 1.8 times that of thiopental, primarily due to the potentiation of the inhibitory action of gamma-aminobutyric acid (GABA) on its GABAa receptor [3, 4].

In dogs, propofol promotes smooth and rapid induction, with loss of consciousness occurring around 60 s after intravenous (iv) administration. This rapid onset is due to its high lipid solubility and quick distribution to the central nervous system (CNS) [1, 5]. In addition, propofol has a minimal cumulative effect on the body, enabling rapid anesthetic recovery once continuous infusion ceases [6].

Depending on the induction dose, propofol may cause bradycardia and a 15%–30% reduction in systolic blood pressure (SBP), diastolic blood pressure (DBP), and mean arterial pressure (MAP). These effects are more pronounced with association to opioid analgesics, particularly in elderly, hypovolemic patients, and those with limited left ventricular function [1].

Cardiac output (CO), defined as the volume of blood ejected from the left ventricle (LV) into the systemic circulation per unit of time, is a crucial hemodynamic variable for monitoring anesthetized dogs or those in intensive care [7]. It provides a reliable therapeutic guide regarding tissue oxygen supply and demand in various hemodynamic states [8].

Methods for measuring CO include invasive and noninvasive techniques [9]. Thermodilution, considered the gold standard, involves inserting a catheter into the pulmonary artery [10]. However, this method carries risks such as arrhythmias, vascular injuries, infections, and even systematic errors related to operator's experience, hemodynamic status, and patient's clinical condition [11].

In this context, echocardiography, either minimally invasive (transesophageal) or noninvasive (transthoracic), represents a valid alternative to thermodilution [12]. The literature lacks consensus on the most effective method, and obtaining satisfactory results depends on the operator's experience in manually delimiting ventricular borders and correctly identifying cardiac structures during echocardiography [9, 13, 14]. Adequate analysis and interpretation of results are also crucial [15, 16].

Then, the transthoracic echocardiography is a viable and effective alternative for measuring hemodynamic indices, presenting lower risk and greater patient comfort compared with thermodilution. This noninvasive technique has a high correlation in data accuracy [17].

Given the above, the study aimed to evaluate whether the isolated use of propofol during anesthetic induction in dogs affects hemodynamic variables assessed through echocardiography. We hypothesized that propofol use would reduce the hemodynamic variables in healthy dogs after anesthetic induction.

## 2. Materials and Methods

### 2.1. Animals

The study was approved by the local university ethics committee (CEUA, no 704/2020). It included 12 male and female dogs of various breeds, aged one to three years, and weighing between 7 and 14 kg. All dogs would be anesthetized for the elective sterilization procedure.

Inclusion criteria required that the dogs be healthy, classified as ASA I (American Society of Anesthesiologists), with normal general physical examination, echocardiographic and electrocardiographic examination, and laboratory tests. These tests included blood count, serum biochemistry (alanine aminotransferase, albumin, and creatinine), and negative serology for Leishmaniasis (indirect ELISA for detecting anti-Leishmania IgG antibodies). In addition, an Informed Consent Form (ICF) signed by their owners was mandatory.

Dogs were excluded if they had abnormal examination results, positive serology for Leishmaniasis, were classified as obese or pregnant, or lacked a signed ICF.

The experiment was conducted at the Laboratory of Anesthesia and Experimental Surgery at the São Paulo State University (UNESP), School of Veterinary Medicine of Araçatuba (FMVA).

### 2.2. Experimental Protocol

The dogs underwent a 12-h food fast and a 2-hour water fast. They were weighed, and their thoracic region (between the third and fifth intercostal space on the right side) was shaved for echocardiographic examination. The dorsal region of the thoracic limbs was shaved for cephalic vein catheterization. Heparin solution was administered to the catheter after obtaining venous access. Then, the animals were conditioned in a temperature-controlled environment at 23°C for 15 min to reduce stress.

No preanesthetic medications or other drugs were administered before collecting the hemodynamic variables, in order to avoid potential pharmacokinetic and pharmacodynamic interferences.

Anesthetic induction was performed using propofol (Propotil® Midfarma Produtos Farmacêuticos Ltda, Mandaguaçu, PR, Brazil) administered with a syringe pump (MedRena SP50 Vet, Shenzhen, China) at a rate of 3 mg/kg/minute. Induction was continued until the loss of eyelid and laryngotracheal reflexes, mandibular tone, and eye rotation. The time required was measured, and no coinducers were used. Induction and its interruption were performed by the same anesthetist.

Echocardiographic evaluations were conducted at two different times: before anesthetic induction (MB), while the dogs were awake, and immediately after induction interruption (MI), before orotracheal intubation, with the dogs positioned in right lateral decubitus on a mattress suitable for echocardiographic assessment.

### 2.3. Echocardiographic Evaluation

The volumes of the LV at the end of diastole (LVVd) and systole (LVVs) were measured using Simpson's uniplanar method in the four-chamber longitudinal view, performed immediately before and after induction. This allowed the calculation of the systolic volume (SV) and ejection fraction (EF%) of the LV. Heart rate (HR), Doppler Ejection Index (DEI), and Doppler Cardiac Index (DCI) were subsequently calculated using the following formulas: DEI = SV/body surface area (BSA), when BSA = (weight × 0.67)/1000, and DCI = (DEI × HR)/1000 [18, 19].

A Doppler echocardiography machine (Esaote Mylab TM 30 VET Gold) with multifrequency phased array transducers, ranging from 1 to 4 MHz and 3 to 8 MHz, along with simultaneous electrocardiographic monitoring, was used. During anesthetic induction, the dogs were positioned in right lateral decubitus, allowing access to the right parasternal window to obtain echocardiographic images. Then, cineloops of cardiac cycles were recorded and stored for later analysis. The same experienced operator (GAB) was responsible for the acquisition and analysis of the echocardiographic variables of interest. All measurements were performed in triplicate to calculate the average of three cardiac cycles.

### 2.4. Statistical Analysis

The normality of the studied variables was assessed using a Q-Q plot, which compared the quantiles of the residuals with those of a theoretical normal distribution. The linearity of the points suggested that all hemodynamic variables in the study were normally distributed. With this assumption met, the variables were subjected to a paired *t*-test, comparing the means before and after induction with propofol (*p* value < 0.05). Data analysis was performed using the R v.4.1.0 software (R CORE TEAM, 2021).

## 3. Results

Twelve mixed-breed dogs, including nine females and three males, aged 1–3 years, were subjected to the experimental protocol. The dogs had a mean weight of 10.2 ± 2.4 kg (mean ± sd). The mean dose of propofol used for induction was 7.49 ± 1.22 mg/kg (mean ± sd), and the induction time average was 150 ± 23 s (mean ± sd) (Table 1).

Table 2 presents the mean and standard deviation of the echocardiographic variables. A significant reduction was observed in the mean EF% and DEI values before and after induction with propofol in healthy dogs.

## 4. Discussion

Under the study conditions, anesthetic induction with propofol resulted in a reduction in EF (%) and DEI, without significant changes in DCI and HR in healthy dogs.

The short period of time between the end of anesthetic induction and the immediate orotracheal intubation of the dogs limited the acquisition of other hemodynamic variables, such as blood pressure measurement, and a complete echocardiographic study. On the other hand, echocardiography provides noninvasive and rapidly acquired tools that allow obtaining morphofunctional information of the myocardium.

CO is a crucial hemodynamic component defined as the volume of blood the heart propels per unit of time, quantified by measuring flow (L/minute). It is calculated as the product of SV and HR: CO = SV × HR. Different CO values can occur due to size variations among animals of the same specie [18–20]. Thus, proper standardization and comparison of CO values are achieved by indexing the obtained value (L/minute) by the BSA, resulting in the DCI, expressed in L/minute/m^2^ [21].

In our study, DCI did not demonstrate significant difference between the moments evaluated. In another study, where dogs were premedicated with acepromazine (0.015 mg/kg) and methadone (0.15 mg/kg), DCI did not show a significant difference after induction with propofol (5 mg/kg for 30 s). This fact was linked to the maintenance of CO due to a positive chronotropic response, probably compensating the reduction in the MAP [22]. However, the inclusion of preanesthetic medications may limit the interpretation of the real effect of anesthetic induction with propofol. Therefore, in our study, we chose to perform anesthetic induction only with propofol, in order to evaluate echocardiographic changes without interference from other pharmacological agents.

In addition, HR and SV did not demonstrate significant differences between MB and MI. The stroke volume, amount of blood ejected from the ventricle during systole, is influenced by preload, contractility (inotropism), and afterload [23]. In our study, there was no significant difference between LVVd and LVVs, a fact that may suggest an absence of preload increase and maintenance of contractility. Hemodynamic studies performed with the aid of echocardiography suggest a possible negative inotropic effect of propofol on the myocardium [24], which may justify the significant reduction in EF% and DEI between MB and MI.

Conversely, it has been shown that the direct negative effect of propofol on the heart is relatively small at clinical concentrations; however, preanesthetic medications were included in association with propofol, which makes it impossible to evaluate the hemodynamic effects of propofol alone [22].

Therefore, the significant reduction in EF% and DEI may be related to a decrease in systemic vascular resistance promoted by the inducing agent [25], leading to reduced venous return and consequent reduction of these echocardiographic indices that represent left ventricular ejection. Unfortunately, variables that allow assessment of vascular resistance after propofol administration were not measured in our study. Therefore, further studies should be conducted to demonstrate the effects of anesthetic induction with propofol alone on these hemodynamic variables.

EF% measures the percentage of volume ejected from the LV during systole [26]. Like CO, a more reliable way to evaluate the fraction of blood ejected by the LV is through indexing, resulting in the DEI, expressed in mL/beat/m^2^ [20]. We must consider that, even without demonstrating a significant difference, there was an increase in HR in MI. This fact may lead to a reduction in diastolic time, culminating in a reduction in SV. This would also explain the significant decrease in the value of EF% and DEI and the maintenance of DCI observed in our study [23].

Our study has limitations, including the lack of blood pressure measurements before and after anesthetic induction, the small sample size, and the absence of parameters to evaluate the metabolic demand of anesthetized dogs.

## 5. Conclusions

Anesthetic induction with propofol significantly reduced left ventricular EF% and DEI under the studied conditions. The other echocardiographic parameters remained unchanged, indicating its stability with the use of propofol as an inducing agent at the administered rate used.

## Figures and Tables

**Table 1:** Dose and total volume of propofol and time required for induction per dog (*n* = 12).

**Animal identification**	**Dose (mg/kg)**	**Total volume (mL)**	**Induction time (sec)**
A1	6.01	4.51	125
A2	7.83	8.61	151
A3	5.76	4.87	115
A4	6.67	8.34	134
A5	8.23	8.39	166
A6	7.08	5.13	141
A7	8.60	10.83	172
A8	9.39	12.82	188
A9	7.0	4.9	139
A10	9.46	12.3	185
A11	7.17	7.53	144
A12	6.72	5.58	135

**Table 2:** Hemodynamic and echocardiographic variables (mean ± standard deviation) of dogs (*N* = 12) at the baseline (MB) and immediately after induction (MI) with propofol (3 mg/kg/minute).

**Hemodynamic variable**	**MB**	**MI**	**p** **value**
HR	128 ± 25	132 ± 35	0.07
EF (%)	70 ± 5	65 ± 8	0.01∗
SV (mL/beat)	12.9 ± 3.6	10.9 ± 4.9	0.08
DEI (mL/beat/m^2^)	27.1 ± 4.9	22.4 ± 6.9	0.04∗
DCI (L/minute/m^2^)	3.5 ± 0.9	2.9 ± 0.9	0.10
LVVd (mL)	18.6 ± 5.22	16.9 ± 6.03	0.16
LVVs (mL)	5.65 ± 2.03	5.98 ± 1.95	0.36

*Note:p* value < 0.05 indicates statistical difference.

Abbreviations: DCI = Doppler cardiac index, DEI = Doppler ejection index, EF (%) = ejection fraction, HR = heart rate (bpm), LVVd = left ventricular volume in diastole, LVVs = left ventricular volume in systole, SV = systolic volume.

^∗^Significant difference between MB and MI.

## Data Availability

The data that support the findings of this study are available from the corresponding author upon reasonable request.
